# Supplementary material for: Ventilator-Associated Pneumonia After Cardiac Arrest and Prevention Strategies: A Narrative Review
Source: Medicina (Kaunas). 2025 Jan 5;61(1):78. doi: 10.3390/medicina61010078 (PMC11767168; doi:10.3390/medicina61010078)
Supplement: Supplementary file 1 [file medicina-61-00078-s001.zip › medicina-3365817-supplementary.pdf]

Table S1 High to moderate degree of certainty and Moderate to low degree of certainty

High to moderate degree of certainty	Moderate to low degree of certainty.
Girotra S, Nallamothu BK, Spertus JA, Li Y, Krumholz HM, Chan PS; Trends in survival after in-hospital cardiac arrest. N Engl J Med. 2012 Nov 15;367(20):1912-20. doi: 10.1056/NEJMoa1109148. PMID: 23150959; PMCID: PMC3517894.<br>Jain S, Self WH, Wunderink RG, Fakhran S, Balk R, Bramley AM, Reed C, Grijalva CG, Anderson EJ, Courtney DM, Chappell JD, Qi C, Hart EM, Carroll F, Trabue C, Donnelly HK, Williams DJ, Zhu Y, Arnold SR, Ampofo K, Waterer GW, Levine M, Lindstrom S, Winchell JM, Katz JM, Erdman D, Schneider E, Hicks LA, McCullers JA, Pavia AT, Edwards KM, Finelli L; CDC EPIC Study Team. Community-Acquired Pneumonia Requiring Hospitalization among U.S. Adults. N Engl J Med. 2015 Jul 30;373(5):415-27. doi: 10.1056/NEJMoa1500245. Epub 2015 Jul 14. PMID: 26172429; PMCID: PMC4728150.<br>Wang HE, Abella BS, Callaway CW; American Heart Association National Registry of Cardiopulmonary Resuscitation Investigators. Risk of cardiopulmonary arrest after acute respiratory compromise in hospitalized patients. Resuscitation. 2008 Nov;79(2):234-40. doi: 10.1016/j.resuscitation.2008.06.025. Epub 2008 Aug 8. PMID: 18692287.<br>Khilnani GC, Arafath TK, Hadda V, Kapil A, Sood S, Sharma SK. Comparison of bronchoscopic and non-bronchoscopic techniques for diagnosis of ventilator associated pneumonia. Indian J Crit Care Med. 2011 Jan;15(1):16-23. doi: 10.4103/0972-5229.78218. PMID: 21633541; PMCID: PMC3097537.<br>Vardakas KZ, Mavros MN, Roussos N, Falagas ME. Meta-analysis of randomized controlled trials of vancomycin for the treatment of patients with gram-positive infections: focus on the study design. Mayo Clin Proc. 2012 Apr;87(4):349-63. doi: 10.1016/j.mayocp.2011.12.011. PMID: 22469348; PMCID: PMC3538415.<br>Antonelli M, Conti G, Rocco M, Bufi M, De Blasi RA, Vivino G, Gasparetto A, Meduri GU. A comparison of noninvasive positive-pressure ventilation and conventional mechanical ventilation in patients with acute respiratory failure. N Engl J Med. 1998 Aug 13;339(7):429-35. doi: 10.1056/NEJM199808133390703. PMID: 9700176.<br>Torres A, Serra-Batlles J, Ros E, Piera C, Puig de la Bellacasa J, Cobos A, Lomeña F, Rodríguez-Roisin R. Pulmonary aspiration of gastric contents in patients receiving mechanical ventilation: the effect of body position. Ann Intern Med. 1992 Apr 1;116(7):540-3. doi:	Merchant RM, Berg RA, Yang L, Becker LB, Groeneveld PW, Chan PS; American Heart Association's Get With the Guidelines-Resuscitation Investigators. Hospital variation in survival after in-hospital cardiac arrest. J Am Heart Assoc. 2014 Jan 31;3(1):e000400. doi: 10.1161/JAHA.113.000400. PMID: 24487717; PMCID: PMC3959682.<br>[illegible]gon JY, Chastre J, Hance AJ, Montravers P, Novara A, Gibert C. Nosocomial pneumonia in ventilated patients: a cohort study evaluating attributable mortality and hospital stay. Am J Med. 1993 Mar;94(3):281-8. doi: 10.1016/0002-9343(93)90060-3. PMID: 8452152.<br>[illegible]sslacher J, Steinkohl F, Ulmer H, Lehner G, Klein S, Mayerhoefer T, Joannidis M. Increased risk of ventilator-associated pneumonia in patients after cardiac arrest treated with mild therapeutic hypothermia. Acta Anaesthesiol Scand. 2022 Jul;66(6):704-712. doi: 10.1111/aas.14063. Epub 2022 Apr 10. PMID: 35338658; PMCID: PMC9321159.<br>[illegible]icky PH, Dupuis C, Cerf C, Siami S, Cohen Y, Laurent V, Mourvillier B, Reignier J, Goldgran-Toledano D, Schwebel C, Ruckly S, de Montmollin E, Buetti N, Timsit JF. Ventilator-Associated Pneumonia in COVID-19 Patients Admitted in Intensive Care Units: Relapse, Therapeutic Failure and Attributable Mortality-A Multicentric Observational Study from the OutcomeRea Network. J Clin Med. 2023 Feb 6;12(4):1298. doi: 10.3390/jcm12041298. PMID: 36835834; PMCID: PMC9961155.<br>[illegible]ang Y, Jiao Y, Zhang J, Xu J, Cheng Q, Li Y, Liang S, Li H, Gong J, Zhu Y, Song L, Rong Z, Liu B, Jie Z, Sun S, Li P, Wang G, Qu J; Infection Assembly of Shanghai Respiratory Society. Microbial Etiology and Prognostic Factors of Ventilator-associated Pneumonia: A Multicenter Retrospective Study in Shanghai. Clin Infect Dis. 2018 Nov 13;67(suppl_2):S146-S152. doi: 10.1093/cid/ciy686. PMID: 30423049.<br>[illegible]ing C, Zhang Y, Yang Z, Wang J, Jin A, Wang W, Chen R, Zhan S. Incidence, temporal trend and factors associated with ventilator-associated pneumonia in mainland China: a systematic review and meta-analysis. BMC Infect Dis. 2017 Jul 4;17(1):468. doi: 10.1186/s12879-017-2566-7. PMID: 28676087; PMCID: PMC5496595.

10.7326/0003-4819-116-7-540. PMID: 1543307.

Brook AD, Ahrens TS, Schaiff R, Prentice D, Sherman G, Shannon W, Kollef MH. Effect of a nursing-implemented sedation protocol on the duration of mechanical ventilation. Crit Care Med. 1999 Dec;27(12):2609-15. doi: 10.1097/00003246-199912000-00001. PMID: 10628598.

Berra L, De Marchi L, Yu ZX, Laquerriere P, Baccarelli A, Kolobow T. Endotracheal tubes coated with antiseptics decrease bacterial colonization of the ventilator circuits, lungs, and endotracheal tube. Anesthesiology. 2004 Jun;100(6):1446-56. doi: 10.1097/00000542-200406000-00017. PMID: 15166564.

Karchmer TB, Giannetta ET, Muto CA, Strain BA, Farr BM. A randomized crossover study of silver-coated urinary catheters in hospitalized patients. Arch Intern Med. 2000 Nov 27;160(21):3294-8. doi: 10.1001/archinte.160.21.3294. PMID: 11088092.

Seguin P, Tanguy M, Laviolle B, Tirel O, Mallédant Y. Effect of oropharyngeal decontamination by povidone-iodine on ventilator-associated pneumonia in patients with head trauma. Crit Care Med. 2006 May;34(5):1514-9. doi: 10.1097/01.CCM.0000214516.73076.82. PMID: 16540962.

Morrow LE, Kollef MH, Casale TB. Probiotic prophylaxis of ventilator-associated pneumonia: a blinded, randomized, controlled trial. Am J Respir Crit Care Med. 2010 Oct 15;182(8):1058-64. doi: 10.1164/rccm.200912-1853OC. Epub 2010 Jun 3. PMID: 20522788; PMCID: PMC2970846.

Dahyot-Fizelier C, Lasocki S, Kerforne T, Perrigault PF, Geeraerts T, Asehnoune K, Cinotti R, Launey Y, Cottenceau V, Laffon M, Gaillard T, Boisson M, Aleyrat C, Frasca D, Mimoz O; PROPHY-VAP Study Group and the ATLANREA Study Group. Ceftriaxone to prevent early ventilator-associated pneumonia in patients with acute brain injury: a multicentre, randomised, double-blind, placebo-controlled, assessor-masked superiority trial. Lancet Respir Med. 2024 May;12(5):375-385. doi: 10.1016/S2213-2600(23)00471-X. Epub 2024 Jan 20. PMID: 38262428.

Tavernier E, Barbier F, Meziani F, Quenot JP, Herbrecht JE, Landais M, Roux D, Seguin P, Schnell D, Veinstein A, Veber B, Lasocki S, Lu Q, Beduneau G, Ferrandiere M, Dahyot-Fizelier C, Plantefeve G, Nay MA, Merdji H, Andreu P, Vecellio L, Muller G, Cabrera M, Le Pennec D, Respaud R, Lanotte P, Gregoire N, Leclerc M, Helms J, Boulain T, Lacherade JC, Ehrmann S; REVA network and the CRICS-TRIGGESEP F-CRIN network. Inhaled amikacin versus placebo to prevent ventilator-associated pneumonia: the AMIKINHAL double-blind multicentre randomised controlled trial protocol. BMJ

ozco-Levi M, Torres A, Ferrer M, Piera C, el-Ebiary M, de la Bellacasa JP, Rodriguez-Roisin R. Semirecumbent position protects from pulmonary aspiration but not completely from gastroesophageal reflux in mechanically ventilated patients. Am J Respir Crit Care Med. 1995 Oct;152(4 Pt 1):1387-90. doi: 10.1164/ajrccm.152.4.7551400. PMID: 7551400.

ng K, Chen B, Wang M, Chen D, Hui L, Guo S, Ji T, Shang F. The effect of early mobilization in critically ill patients: A meta-analysis. Nurs Crit Care. 2020 Nov;25(6):360-367. doi: 10.1111/nicc.12455. Epub 2019 Jun 20. PMID: 31219229.

Open. 2021 Sep 14;11(9):e048591. doi: 10.1136/bmjopen-2020-048591. PMID: 34521664; PMCID: PMC8442072.	
